# Evaluation of ultraviolet (UV‐C) light treatment for microbial inactivation in agricultural waters with different levels of turbidity

**DOI:** 10.1002/fsn3.1412

**Published:** 2020-01-14

**Authors:** Achyut Adhikari, Katheryn J. Parraga Estrada, Vijay S. Chhetri, Marlene Janes, Kathryn Fontenot, John C. Beaulieu

**Affiliations:** ^1^ School of Nutrition and Food Sciences Louisiana State University Agricultural Center Baton Rouge LA USA; ^2^ School of Plant, Environmental and Soil Sciences Louisiana State University Agricultural Center Baton Rouge LA USA; ^3^ United States Department of Agriculture Agricultural Research Service New Orleans LA USA

**Keywords:** generic *E.* *coli*, irrigation water, produce safety, surface water, UV‐C light

## Abstract

Produce growers using surface or well water to irrigate their crops may require an appropriate water treatment system in place to meet the water quality standard imposed by FSMA Produce Safety Rule. This study evaluated the potential of using ultraviolet (UV‐C) treatment in reducing the microbial population in agricultural water. Waters with turbidity levels ranging from 10.93 to 23.32 Nephelometric Turbidity Units (NTU) were prepared by mixing pond water and well water. The waters were inoculated with a cocktail of generic *Escherichia* *coli* (ATCC 23716, 25922, and 11775) and then treated with UV‐C light (20–60 mJ/cm^2^). All tested doses of the UV‐C treatment reduced the *E.* *coli* levels significantly (*p* < .05) in the water samples with the turbidity levels up to 23.32 NTU. The decrease in the turbidity from 23.32 to 10.93 NTU increased the level of reduction by more than 2.15 log most probable number (MPN)/100 ml). UV‐C treatment effectively reduces microbial load in agriculture water; however, turbidity of water may significantly affect the disinfection efficacy. The study also demonstrated that sprinkler system resulted in a higher level of contamination of cantaloupes compared with drip irrigation. The results indicated that UV‐C treatment could be a promising strategy in reducing the produce safety risks associated with irrigation water.

## Graphical abstract

This study evaluated the potential of using ultraviolet (UV‐C) treatment in reducing the microbial population in agricultural water. All tested doses of the UV‐C treatment reduced the *E.* *coli* levels significantly (*p* < .05) in the water samples with the turbidity levels up to 23.32 NTU. The decrease in the turbidity from 23.32 to 10.93 NTU increased the level of reduction by more than 2.15 log most probable number (MPN)/100 ml). UV‐C treatment effectively reduces microbial load in agriculture water; however, turbidity of water may significantly affect the disinfection efficacy.

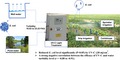

## 1 INTRODUCTION

Produce safety has become the forefront in agricultural issues that address potential public health risks due to an increase in foodborne disease outbreaks (Breitenmoser, Fretz, Schmid, Besl, & Etter, 2011; CDC, 2015; Fan, Annous, Beaulieu, & Sites, 2008; Mazari‐Hiriart et al., 2008). Preharvest environment and farm activities are the major sources of microbial contamination in fresh produce (Chhetri, Fontenot, et al., 2019; Weller et al., 2017). Agricultural water is one of the important vehicles for human pathogens (Cooley et al., 2007; Ijabadeniyi, Debusho, Vanderlinde, & Buys, 2011; Park et al., 2012). If the contaminated water is used for the irrigation of agricultural crops, it poses a high risk of foodborne outbreaks (Olaimat & Holley, 2012).

The Food Safety Modernization Act (FSMA) Produce Safety Rule (PSR) has identified agricultural water as an important source of microbial contamination to produce (Fan et al., 2008). The rule requires that the water used for irrigation and production/processing of fresh produce must be safe and of adequate sanitary quality for its intended use. The growers are recommended to regularly monitor the microbial quality of their water sources by testing generic *E.* *coli* (FDA, 2019; Chhetri, 2018). The easily distinguishable characteristics of generic *E.* *coli* make it a principal indicator organism to assess water contamination with human pathogens (FDA, 2019). It is a predictor of undesirable conditions such as ineffective treatment or fecal contamination (Gekenidis et al., 2018).

Several methods are currently available for the treatment of drinking and irrigation water such as chlorination (Beuchat, 1999; Chhetri, Janes, King, Doerrler, & Adhikari, 2019; Whan et al., 2001), chlorine dioxide (Carrillo, Puente, & Bashan, 1996), ozone (Kim, Yousef, & Dave, 1999), and filtration (Koivunen, Siitonen, & Heinonen‐Tanski, 2003). Conventional chemical treatments may affect the quality of the crops and soil (Hua & Reckhow, 2007) while conventional filtration methods may not be suitable for surface water due to its complexity and variability in the effectiveness. The efficacy of filtrations may vary with raw water quality including turbidity, type of microorganisms, type of filtration material and pore size, and filtration rate (LeChevallier & Au, 2004). Regular cleaning for maintaining adequate water flow rate may be another limitation of filtration system (Burch & Thomas, 1998). The use of UV‐C light may overcome these limitations (Hijnen, Beerendonk, & Medema, 2006). The mechanism of UV‐C light disinfection is based on the formation of pyrimidine dimers in microbial DNA. The dimers interfere with the replication, transcription, and, thus, translation process of microorganisms (Koutchma, Forney, & Moraru, 2009).

In the United States, groundwater, surface water, and municipal water are the common sources of irrigation water for fresh produce (Jongman, Chidamba, & Korsten, 2017; Pedrero, Kalavrouziotis, Alarcón, Koukoulakis, & Asano, 2010). Although chlorine treatment is the most common practice for disinfecting municipal water, UV‐C light treatment has also been applied by some municipalities (Hijnen et al., 2006). As the turbidity of groundwater and surface water is generally higher than municipal water (Topalcengiz, Strawn, & Danyluk, 2017), the efficacy of UV‐C in these waters could be different. This study used a large volume of surface water and well water to simulate a water treatment system in agricultural settings (Jones, Worobo, & Smart, 2014). This study evaluated the efficacy of UV‐C light treatment in reducing generic *E.* *coli* in surface water, well water, and their mixtures with different level of turbidity. Furthermore, the effect of UV‐C‐treated irrigation water on the generic *E.* *coli* levels on the cantaloupes was evaluated in an agriculture setting.

## 2 MATERIALS AND METHODS

### 2.1 Inoculum preparation

A cocktail of generic *E.* *coli* strains (ATCC 23716, 25922, and 11775) was used in this study. These strains were previously used in irrigation water treatment studies and are among the few well‐characterized surrogates for use in field trials (Harris et al., 2012). Before activation, bacterial strains were stored at −80°C. Frozen cultures were activated in three successive passes by following the procedure described by (Adhikari et al., 2015). The final inoculum size of the generic *E.* *coli* was 10^8^ CFU/mL.

### 2.2 Water sample collection

Water samples were collected from a pond and a well located at the LSU AgCenter Botanic Gardens in Baton Rouge, Louisiana. To resemble natural variability that may occur in surface water irrigation sources, we used pond water (P), well water (W), and two mixtures of the pond and well water (PW, 1:1; WP, 4:1). The different sources and mixture of waters helped us to maintain a different level of turbidity and transmittance (Tables 1 and 2). The waters were collected in a tank (1,000 L) in the proportions mentioned above. The pond water was prefiltered using a cloth (Standard Test Sieve No. 25; W.S. Tyler) attached at the end of the pipe to remove larger particles. Each batch of water in the tank (1,000 L) was inoculated with the generic *E.* *coli* followed by agitation for 2 min using a sterile plastic pedal. Water samples (100 ml) were collected from the tank before and after inoculation. The final inoculum size in the water was maintained at 7–8 log MPN/100 ml. Experiments were replicated three times to capture the variability associated with the water and environmental conditions. The pH (Orion™, 2‐Star Benchtop pH Meter, Thermo Scientific™), turbidity (TU‐2016, LT Lutron), and UV light absorbance and transmittance (Beckman Coulter™ DU^®^‐530, GMI‐INC) at 254 nm were measured for all the water samples.

**Table 1:** UV‐C light doses applied to the irrigation water sources

Water source	UV‐C Dose (mJ/cm^2^)									
	10–20	20–30	30–40	40–50	50–60	60–70	70–80	80–90	90–100	120–130
Pond	T	T	T	T	T	T	N/S	T	T	T
Pond + Well (1:1) (PW)	T	T	T	T	T	T	N/S	T	N/S	T
Well + Pond (4:1) (WP)	N/S	T	T	T	T	N/S	T	*N*/S	N/S	N/S
Well	N/S	T	T	T	T	T	T	N/S	T	N/S

Abbreviations: N/S, Not studied; T, Water sample and doses tested.

**Table 2:** pH, turbidity, and percentage of transmission values for water sources

Water^1^	pH	Turbidity [NTU]	Transmission (%)
Pond	7.04 ± 0.11	23.32 ± 2.9	29.16 ± 0.47
PW	7.76 ± 0.62	19.70 ± 5.8	53.74 ± 18.2
WP	8.01 ± 0.11	13.16 ± 3.7	74.57 ± 0.97
Well	8.07 ± 0.06	10.93 ± 2.0	88.11 ± 2.28

Abbreviations of treatments are as follows: Pond, pond water; PW, pond water + well water (1:1); Well, well water; WP, well water + pond water (4:1).

### 2.3 UV‐C light treatment

A UV‐C light treatment equipment (PMD 150C1/4, Aquionics, Slough) was used in this study. The PMD 150C1/4 uses a photon medium‐pressure disinfection with an arc tube that has a medium‐pressure UV germicidal lamp (253.7 nm) and a UV chamber (0.2 m diameter) consisting of a 3.4 kW Lamp. The UV irradiance (mJ/cm^2^), the temperature of the system (˚C), and transmission (%) were measured at the time of treatment. The UV inactivation rate was calculated based on the log reduction in generic *E.* *coli*. Preliminary experiments with generic *E.* *coli* inoculated water were performed at different doses (10–20, 20–30, 30–40, 40–50, 50–60, 60–70, 80–90, 90–100, and 120–130 mJ/cm^2^) (Table 1) and four best ranges of UV doses (20–30, 30–40, 40–50, and 50–60 mJ/cm^2^) were selected for this study. Each batch of inoculated water was passed through the UV‐C treatment equipment at different doses and was collected in another tank. Water samples (triplicate) were collected in a sterilized container (100 ml) before and after each UV treatment. All the experimental treatments were repeated three times.

### 2.4 Microbiological examination of Irrigation water

Generic *E.* *coli* was quantified using Quanti‐Tray 2000‐Colilert^®^ (IDEXX Laboratories). Water samples (100 ml) were mixed with Colilert^®^ medium for 60 s until the medium was completely dissolved, and appropriate decimal dilutions were made based on the treatment. The Quanti‐trays were incubated at 35°C for 18–24 hr. Results were enumerated by counting wells for total coliforms (yellow colored) and *E.* *coli* fluorescence under a portable fluorescent UV lamp (WL200, Hanovia LTD, Aquionics, UK). The results were expressed as the most probable number (MPN) using a chart provided by IDEXX. Pathogens (*E.* *coli* O157: H7 and *Salmonella* spp.) present in water samples were examined by using the immunomagnetic separation (IMS) technique using BeadRetriever^TM^ (Thermo Scientific). Briefly, 18 ml of water sample was mixed with 160 ml TSB and incubated for 24 hr at 37°C. Then, IMS was performed for EPEC/VTEC using 1ml of the pre‐enriched water sample with 10 μL Dynabeads anti‐ *E.* *coli* O157 (Invitrogen Dynal, AS, Oslo, Norway) following the manufacturer's instructions. Similarly, for *Salmonella* spp., 18 ml of water sample was pre‐enriched in 160 ml of buffer peptone water (BPW) at 37°C for 18–24 hr. Then, IMS was performed using 1 ml of pre‐enrich BPW with Dynabeads (10 μL) anti‐*Salmonella* (Invitrogen Dynal, AS) following the manufacturer's instruction. Confirmation test involved *E.* *coli* O157 latex agglutination (Oxoid) and *Salmonella* Latex Test (Remel Europe Ltd., Wellcolex^®^, Thermo Scientific™).

### 2.5 Generic *E.* *coli* microstructure by Scanning Electron Microscopy

Scanning electron microscopy (*SEM*) was used to examine the microstructure of generic *E.* *coli* in the pond water using the method previously described by Kenzaka and Tani (Kenzaka & Tani, 2012). Briefly, the samples were centrifuged at 10,000*g* for 5 min, and the pellets were suspended in phosphate‐buffered saline (PBS; pH 7.2). The suspension was passed through a 0.45 μm filter to bind particles present in the water. The filter was dehydrated using an ethanol gradient (70, 80, 95, and 99% v/v, 30 min in each and 2× with 99%), and critical‐point‐dried in a DCP‐1 critical point drying apparatus (Denton Vacuum, Inc.). The filter was mounted on aluminum stubs with double‐stick tape and sputter‐coated with a platinum layer of ~10 nm (Leica EMS550X). *SEM* was performed using a high‐vacuum scanning electron microscope (JSM‐6610LV, Jeol Ltd.), at 10 kV.

### 2.6 Irrigation of cantaloupes with UV‐C‐treated water

**Figure 1:**
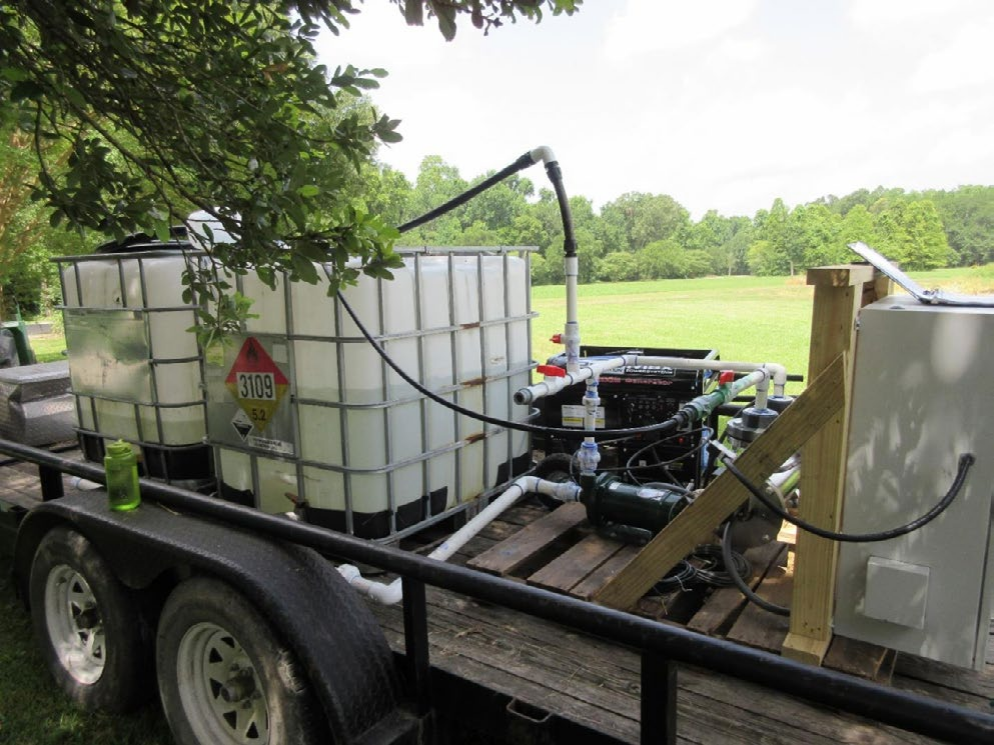
UV‐C treatment system (PMD 150C1/4, Aquionics, Slough, UK) for irrigation water. LSU AgCenter Botanical Garden, Baton Rouge, Louisiana

“Hales Best Jumbo” cantaloupe seedlings (*Cucumis* *melo* reticulatus) were transplanted in a field at LSU AgCenter Botanic Garden, Baton Rouge, Louisiana. The field had plot sizes of 5′ × 10′ and 10′ × 15′ (3 plots each treatment) with 5 and 10 plants in each plot, respectively. The study was conducted twice, in July and October. The treatment schemes were drip, and sprinkler irrigation with UV‐C‐treated and UV‐C‐untreated water. The irrigation water was prepared by mixing the pond water with well water in an equal proportion (final turbidity: 19.70 NTU). The total volume of the water was 1,000 L in a plastic tank with a capacity of 1,100 L. The water was then inoculated with three generic *E.* *coli* strain cocktail (ATCC 11775, 23716, and 25922) maintaining the final concentration of 7–8 log MPN/100 ml. The water was treated with the UV‐C dose of 50–60 mJ/cm^2^ (Figure 1), which reduced the *E.* *coli* count to 1.74–2.45 log MPN/100 ml. The inoculated but untreated water was used as the control. The study was conducted during two harvesting times, July and October. Irrigation was performed for 20 min on each of the three consecutive days before harvest. After harvesting, cantaloupes were aseptically transferred into sterile plastic bags using gloves and immediately transported to the laboratory maintaining 4°C. An aliquot of 200 ml of peptone water (0.1%) was added to each bag, and the fruits were hand‐massaged for 5 min to dislodge any microorganisms from the fruit surfaces. The supernatant obtained after massaging was used for generic *E.* *coli* analysis using the Colilert Quanti‐Tray method (IDEXX) as previously mentioned.

### 2.7 Statistical analysis

Data were analyzed using the SAS^®^ program (SAS Institute) with an ANOVA analysis, using a Tukey's honestly significant separation difference with a confidence level of 95% (*p* ≤ .05) for every water mixture and each range of dose used.

## 3 RESULTS AND DISCUSSION

### 3.1 Water quality

The pH, turbidity, and transmittance in water samples are shown in Table 2. The pond water and the well water had a pH of 7.04 and 8.07, respectively. The mixtures of pond water and well water, PW (1:1), and WP (4:1) had the pHs of 7.76 and 8.01, respectively. The turbidity of the pond water, well water, PW, and WP was 23.32, 10.93, 19.70, and 13.16 NTU, respectively. The transmission value was lower for the pond water (29.16) compared with the well water (88.11).

The turbidity and pH of the water may vary with sources and season. The US EPA recommends the pH range of irrigation water to be in the range of 6.5–8.4 (USEPA, 2012). We observed that the pHs of the waters that were used in this study were within the EPA range. The turbidity of irrigation water is recommended to be ≤2 NTU (USEPA, 2012). However, if the irrigation water sources are groundwater and surface water, the turbidity may be higher. Topalcengiz et al., 2017 evaluated the quality of the pond water, which have been used for agricultural purpose, from 2012 to 2014 in an area of West Central Florida. They observed the turbidity of the water samples ranging from 1 to 129 FAU (Formazin Attenuation Units) and the pH ranging from 5.08 to 10.7. Sengupta et al., 2012 observed the turbidity of irrigation water in the range of 42 to 183 NTU in Ghana, and during dry seasons, the turbidity of the water increased up to 791 NTU. Our study included four different turbidity levels of water to represent the variations in water turbidity levels. We also analyzed the pond water samples for the presence of *E.* *coli* O157: H7 and *Salmonella* spp. None of the samples was positive for these pathogens.

### 3.2 UV‐C light was effective in reducing the generic *E.* *coli* levels in all the water sources

The effect of UV‐C light treatment in inactivating generic *E.* *coli* in water with different turbidity levels is shown in Table 3. The UV‐C dose of 20–30 mJ/cm^2^ resulted in the reduction in generic *E.* *coli* population by more than 7 log MPN/100 ml in well water (10.93 NTU) and WP (13.16 NTU). The majority of the samples had a bacterial level below the detectable limit of the test (<1 MPN/100 ml). The UV‐C dose of 20–30 mJ/cm^2^ resulted in the reduction in the *E.* *coli* population by 5.35 log MPN/100 ml in PW (19.70 NTU). An increase in UV‐C dose up to 50–60 mJ/cm^2^ did not significantly increase the reduction level. For the pond water (23.32 NTU), the reduction was by 3.75 due to UV‐C dose of 20–30 mJ/cm^2^. An increase in the dose to 50–60 mJ/cm^2^ resulted in a significantly (*p* < .05) higher level of reduction (by 5 log MPN/100 ml). In our preliminary study, we evaluated the UV‐C dose up to 120–130 mJ/cm^2^ for the pond water and PW to see whether the bacterial reduction could further be increased. However, we did not see significant changes in the reduction levels (data not shown). Based on this result, we selected the UV‐C doses of 20–60 mJ/cm^2^ in this study.

**Table 3:** Reduction of generic *Escherichia* *coli* in the four water sources using different UV‐C doses^a^

Water source^b^	UV‐C dose (mJ/cm^2^)^c^			
	20–30	30–40	40–50	50–60
Well	7.12 (0.39)^aA^	7.25 (0.39)^aA^	7.24 (0.39)^aA^	7.15 (0.39)^aA^
WP	7.02 (0.34)^aA^	7.13 (0.39)^aA^	7.46 (0.39)^aA^	7.19 (0.39)^aA^
PW	5.35 (0.24)^bA^	5.76 (0.23)^bA^	5.67 (0.23)^bA^	5.65 (0.39)^bA^
Pond	3.75 (0.20)^cB^	3.51 (0.21)^cAB^	3.99 (0.21)^cAB^	5.00 (0.39)^bA^

a Reduction in generic *E.* *coli* values expressed as mean (standard error) in log MPN/100 ml; the initial count was 7 to 8 log MPN/100 ml

b Abbreviations of treatments are as follows: Pond: pond water; PW: pond water + well water (1:1); WP: well water + pond water (4:1); Well: well water.

c UV‐C light doses (mJ/cm^2^) used for the different water sources. Means separated within each vertical column (UV‐C doses) followed by different lowercase letters (a–c) are significantly different (*p* < .05) from each other. Within a row, means followed by different uppercase letters (A–B) are significantly different (*p* < .05); The correlation between the bacterial reduction by UV‐C and turbidity of waters, *r* = −.98 (20–30, 40–50 & 50–60 mJ/cm^2^) and *r* = −.96 (30–40 mJ/cm^2^). The correlation between the bacterial reduction by UV‐C and transmission: *r* = −.91 (20–30 & 50–60 mJ/cm^2^) and *r* = −.88 (30–40 & 40–50 mJ/cm^2^).

**Figure 2:**
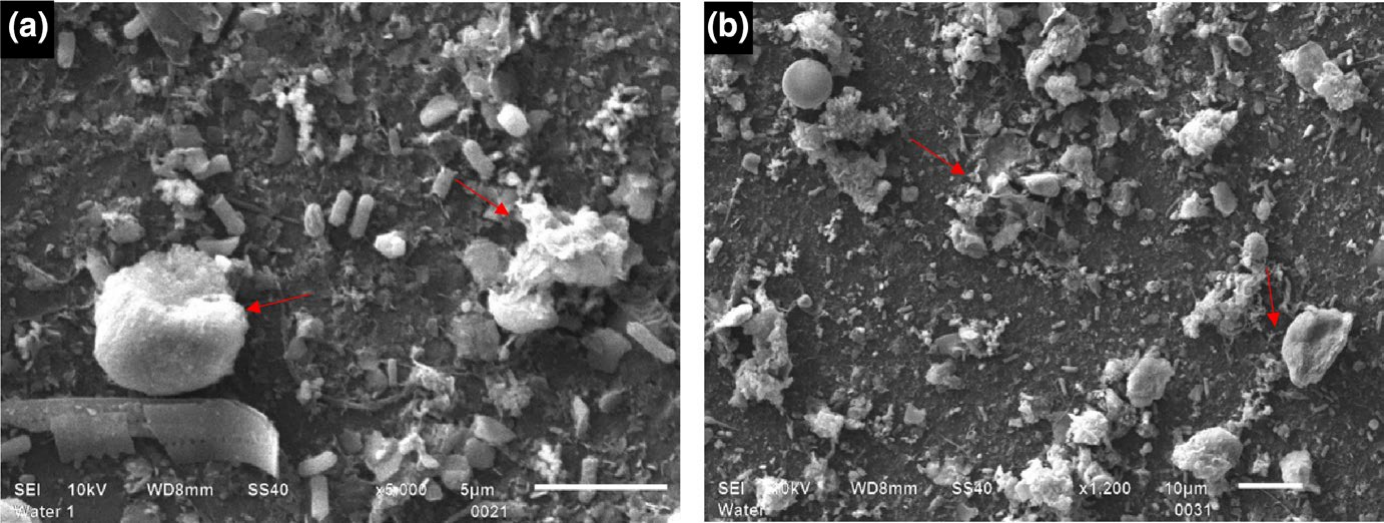
Characterization of generic *Escherichia* *coli* in water by scanning electron microscopy. (a) Particles present in pond water (SEI 10 KW, ×5,000, 5 µm); (b) Bacterial cell (*E.* *coli*) hidden behind suspended particles (SEI 10 KW, ×1,200, 10 µm)

We observed a strong negative correlation between the efficacy of UV‐C treatment (bacterial population reduction) and UV‐C transmission (*r* = −.88 to −.91) and the turbidity level (*r* = −.96 to −.98) of the tested water samples. This indicated that the efficacy of the UV‐C decreased with an increase in the turbidity of water. High levels of turbidity in water could affect the transmittance of UV light, influencing the efficacy of the UV‐C treatment (Jones et al., 2014; Koutchma et al., 2009). The suspended particles in water can absorb and scatter the UV light, subsequently reducing disinfection efficacy (Andreadakis, Mamais, Christoulas, & Kabylafka, 1999; Jolis, Lam, & Pitt, 2001). Scanning electron micrographs showed suspended particles in the pond water (Figure 2a), which were larger than the bacterial size. No significant changes in the efficacy of the treatment with the increase in UV‐C dose in PW and pond water may be attributed to the shielding effect of suspended particles (Cantwell & Hofmann, 2011). Also, the particles might have offered protection to embedded generic *E.* *coli* from UV‐C exposure (Figure 2b), regardless of the level of dose, reducing the efficacy of the treatment. Nonetheless, reduction in *E.* *coli* level by 5 log MPN/100 ml in pond water with the turbidity level of ~23 NTU indicated that the UV‐C could be a promising tool in reducing food safety risks associated with agricultural waters including pond water.

### 3.3 Role of irrigation on the level of *E.* *coli* on cantaloupe surfaces

The level of *E.* *coli* on cantaloupe surfaces after irrigation for three consecutive days is shown in Table 4. In the first harvest (July), the level of *E.* *coli* was up to 3.99 log MPN/cantaloupe and 2.21 log MPN/cantaloupe on sprinkler‐irrigated cantaloupes and drip‐irrigated cantaloupes, respectively. The *E.* *coli* count was higher in the second harvest (October), with up to 5.65 log MPN/cantaloupe in sprinkler‐irrigated cantaloupes and up to 3.70 log MPN/cantaloupe on drip‐irrigated cantaloupes. The counts were significantly different (*p* < .05) between the irrigation systems. However, there was no significant difference between the cantaloupes irrigated with UV‐C‐treated water and the cantaloupes irrigated with untreated water.

**Table 4:** Generic *E.* *coli* levels on the cantaloupe surfaces as affected by UV‐C light treatment and irrigation system

Treatment	Log MPN/cantaloupe			
	First Harvest		Second Harvest	
	Sprinkle	Drip	Sprinkle	Drip
UV‐C	3.15 ± 0.39^aA^	2.21 ± 1.42^aA^	5.20 ± 0.29^qQ^	2.59 ± 1.71^qR^
Control	3.99 ± 0.39^aA^	1.45 ± 1.51^aB^	5.65 ± 0.09^qQ^	3.70 ± 0.94^qR^

Note

The UV‐C treatments dose was 50–60 mJ/cm^2^; the control water was inoculated with generic *E.* *coli* but was not treated. *E.* *coli* levels are expressed as mean ± standard error in log MPN/cantaloupe. First harvest was done in July, and the second harvest was done in October; the harvest was done at 48 hr from last irrigation. Means within each vertical column followed by common lowercase letter (a) are not different (*p* ≥ .05). Within a row (within the harvest), means followed by different uppercase letters (A–B) are significantly different (*p* < .05).

Differences in *E.* *coli* level between the first harvest (July) and the second harvest (October) indicated that season may be associated with the microbial quality of produce items. Other studies also demonstrated variations in microbial population on produce with the season. Ailes et al. (2008) observed higher *E.* *coli* populations on fresh produce in fall compared with spring and winter. While Denis, Zhang, Leroux, Trudel, and Bietlot (2016) observed variations in microbial contamination of cantaloupes between the years, from 2010 to 2012. The number of contaminated cantaloupes was higher in December, September, and June in 2010, 2011, and 2012, respectively (Denis et al., 2016). This seasonal variation in the microbial population may be attributed to the changes in weather conditions such as temperature, humidity, and day length (Ailes et al., 2008; Chhetri, Fontenot, et al., 2019; Chhetri et al., 2018). However, further study is needed to establish relationships between year‐round weather conditions and microbial quality of produce.

Our results indicated that sprinkle irrigation resulted in higher microbial contamination on cantaloupe surfaces compared with drip irrigation. Other studies have reported similar results. Sprinkle irrigation increased the risk of microbial contamination of lettuce surfaces (Fonseca, Fallon, Sanchez, & Nolte, 2011; Van der Linden et al., 2013). Sprinkler irrigation delivers water directly to the surface of crops. If the irrigation water is contaminated, this method becomes an easy mechanism by which pathogens are disseminated on agricultural crops. Also, the sprinkler irrigation may produce injuries on the crop surfaces during harsh environmental conditions creating a favorable condition for the survival of *E.* *coli* (Harapas, Premier, Tomkins, Franz, & Ajlouni, 2010). Drip irrigation has been reported to be comparatively a safe method in terms of cross‐contamination of agriculture crops (Song, Stine, Choi, & Gerba, 2006). A similar effect on the microbial load on cantaloupes between UV‐C‐treated and UV‐C‐untreated water indicated that there could be other potential sources of *E.* *coli* contamination, which greatly dominated the prevalence of *E.* *coli* level on cantaloupes. Contamination of fresh produce can also be attributed to other factors such as soil, via animals or insects, and subsequent human handling (Gutierrez‐Rodriguez & Adhikari, 2018; Harriset al., 2003). Therefore, contamination of produce with *E.* *coli* in farm may be unavoidable, and the presence of generic *E.coli* may not necessarily confirm the presence of pathogens. However, this study suggested that UV‐C treatment can reduce the pathogen levels in irrigation water reducing the produce safety risks associated with irrigation water. Further study is needed using more specific bacterial strains that can be discriminated from the normal environmental flora for a better understanding of the effect of irrigation water quality on crop contamination.

Overall, our study demonstrated that UV‐C treatment could be a promising tool to reduce the microbial load in agriculture water, including surface water. The UV‐C disinfection efficacy was dependent on the turbidity of the water. Therefore, the efficacy of the treatment may be enhanced including a prefiltration step in the treatment system. Our on‐farm study demonstrated that sprinkler system could result in a higher level of contamination of produce compared with drip irrigation. Further study is needed to understand the mechanism to produce contamination through irrigation.

## CONFLICT OF INTEREST

The above‐named authors of the submitted manuscript confirm no conflict of interest.

## ETHICAL STATEMENT

This study does not involve any human or animal testing. The protocol was reviewed and approved by the Inter‐Institutional Biological and Recombinant DNA Safety Committee.
